# Assessment of Cytokine Release against Oral Mucosal Cell Line Culture (TR146) Stimulated by Neutrophil Elastase Associated with Behcet's Disease

**DOI:** 10.1155/2019/6095628

**Published:** 2019-05-26

**Authors:** Azeem Hussain Soomro, Erum Khan, Shafaq Noori, Mohid Abrar Lone, Zahid Syal, Sabir Sheikh

**Affiliations:** ^1^Department of Oral Pathology, Dow University of Health Sciences, Karachi, Sindh, Pakistan; ^2^Department of Oral Pathology, Liaquat University of Medical and Health Sciences, Jamshoro, Sindh, Pakistan; ^3^Department of Biochemistry, Muhammad Bin Qasim Medical College, Karachi, Sindh, Pakistan; ^4^Department of Oral Pathology, Sindh Institute of Oral Health Sciences, Jinnah Sindh Medical University, Karachi, Pakistan

## Abstract

**Aim:**

Cytokines and chemokines may be involved in the onset of oral ulcer in Behcet's disease. The aim of our study is to assess the cytotoxic effects of proinflammatory cytokines and chemokines on reconstructed oral mucosal cell line (TR146) when treated with different concentrations of neutrophil elastase (NE).

**Objective:**

For this purpose, a culture of the oral mucosal model (OMM) prepared from a cell line derived from an oral squamous cell carcinoma of buccal mucosa (TR146) is treated with different concentrations of neutrophil elastase. The cultures were incubated for 4- and 24-hour intervals and designed as follows: culture + artificial saliva served as the negative control; culture + 0.01% SLS (sodium lauryl sulphate) served as the positive control; and culture + NE (10, 50, 100, and 200 nM) served as the treated group.

**Materials and Methods:**

We used sandwich ELISA technique to isolate IL-1*β* (interleukin 1*β*), IL-8, and TNF-*α* (tumor necrosis factor).

**Results:**

We found no significant level of IL-8 and TNF-*α* when treated with different concentrations of neutrophil elastase after 4- and 24-hour incubation. The IL-1*β* level was slightly higher when treated with 100 and 200 nM NE after 24 hours of incubation although a significantly high level was observed at 100 nM NE after 4 hours of incubation. Hence, we found an increase in the level of IL-1*β* when stimulating the reconstructed oral mucosal model (OMM) with different concentrations of NE. This is a preliminary *in vitro* study; however, further research is required to evaluate the cytotoxic effects of cytokines and chemokines released when treated with NE. Moreover, high concentrations of NE are recommended to stimulate the release of cytokines and chemokines against the OMM.

## 1. Introduction

A multisystemic complex disorder of aphthous stomatitis, genital ulceration, and iritis is defined as Behcet's disease (BD) [1]. Patients with BD depicted a significantly higher risk of leukemia, lymphoma, oropharyngeal cancer, thyroid cancer, and prostate cancer [2]. There is no study in which malignant transformation of BD into oral squamous cell carcinoma has been reported. Therefore, we included oral squamous cell carcinoma cell line in the study and assessed cytokines released against the oral mucosal cell line culture stimulated by neutrophil elastase associated with BD. Evaluating the effects of neutrophil elastase in BD using OSCC cell line makes this study novel. It is an immune-mediated systemic vasculitis and characterized by recurrent oral ulcerations, recurrent genital ulceration, skin lesions uveitis [3], rheumatoid arthritis [4], Crohn's disease, and systemic lupus erythematosus (SLE) [5]. Nervous system relapse [6] and vascular disease leading to ocular inflammation are the most common consequences of BD [7]. Epidemiological studies showed a high frequency of this disease in Turkish, Iraqi, Iranian, Korean, and Japanese populations. Prevalence of this disease is more common in men than in women with a ratio of 2–10 : 1 [8]. Most probable effector mechanisms suggested are HLA-B51 antigen-implicated T lymphocyte homeostasis [9]; HLA-Cw 1602 gene marker identification [10]; using infectious agents such as parvovirus B19, *Streptococcus* spp. (e.g., *S. sanguis*, *S. faecalis*, *S. pyogenes*, and *S. salivarius*), *Helicobacter pylori*, *Borrelia burgdorferi*, human herpes simplex virus-6 (HSV-6), and hepatitis A, B, C, and E viruses [11]; using *α*-enolase protein as a target protein for serum anti-endothelial cell antibodies (AECAs) [12], heat shock proteins [13], and autoantigens; stimulation of B cells having cardiolipin antibodies [14]; derangement of endothelial dysfunctions; inducing oxidative stress and imbalance in coagulation and fibrinolytic profiles; and derangement of neutrophils and monocytes [15].

Generally, inflammatory responses are regulated by active serine proteases by processing growth factors, surface receptors, and signaling molecules. Neutrophil elastase from neutrophil serine proteases (NSPs) resides in neutrophil granules and monocyte lysosomes [16]. Neutrophil elastase is a serine protease and is known as a mediator of vascular and tissue injury. However, its inappropriate release has been associated with inflammatory diseases including chronic obstructive pulmonary disease, reperfusion injury, genital tract inflammation, and inflammation of bowel [17]. Numerous studies demonstrated the protective effect of NE inhibitors against neutrophil-mediated tissue injury [18].

Neutrophil hyperactivity in BD patients has been studied previously [19]. Neutrophil elastase concentration is high in BD patients and causes tissue injury; therefore, the aim of the present study is to evaluate the damage of epithelial cells after 4- and 24-hour incubation by measuring cytokines IL-1*β* and IL-8 and TNF-*α* released when stimulating reconstructed oral mucosal model cell lines with different concentrations of neutrophil elastase (NE).

## 2. Materials and Methods

Cultures were obtained from SkinEthic (Episkin, 4, rue Alexander Fleming 69366-Lyon Cedex 7, France), and 0.5 cm^2^ epithelium was reconstituted by an airlifted culture of transformed human keratinocytes for 12 days in a chemically defined medium on inert polycarbonate filters (thickness of the culture at day 5 was 135 *μ*m (indicative value)).

Cultures of the oral mucosal model (OMM) (prepared from a cell line derived from an oral squamous carcinoma of buccal mucosa (TR146)) were purchased from SkinEthic Laboratories (Episkin, 4, rue Alexander Fleming 69366-Lyon Cedex 7, France). SkinEthic cultures were shipped on agar, and on arrival, the maintenance media and cultures were allowed to reach room temperature for one hour; after that, cultures and media were transferred into a laminar flow hood. 500 *μ*l of the maintenance medium was transferred into each well of a 24-well plate, and cultures were removed from the agar plate using sterile tweezers that had been washed and stored in 70% ethanol. Each culture insert was blotted onto a filter paper to get rid of any residual agar, and then the insert was transferred into the 24-well plate containing the maintenance medium and was placed into an incubator at 37°C, 5% CO_2_ in a humidified atmosphere overnight.

## 3. OMM Culture and Treatment

The OMM culture is treated as follows:

OMM + 200 *µ*l of artificial saliva (artificial saliva was obtained from AS Orthan Company) served as the negative control. OMM + 200 *µ*l of 0.01% SLS (0.1 g SLS in 10 ml phosphate buffer) served as the positive control. The OMM treated with 10 nM, 50 nM, 100 nM, and 200 nM NE served as the test group. All the culture supernatants were placed into 6-well plates containing 1 ml of the fresh maintenance medium and subsequently treated with artificial saliva, 0.01% SLS, and 10, 50,100, and 200 nM neutrophil elastase and incubated for 4 and 24 hours at 37°C. After 24 hours, the underlying medium was collected and stored at −80°C for analysis.

## 4. Detection of Cytokines

The levels of IL-8, IL-1*β*, and TNF-*α* in the culture supernatants were measured by commercial enzyme-linked immunosorbent assays (sandwich ELISA technique) specific to cytokines. The culture supernatant samples were used undiluted. The cytokine concentrations in the sample were determined by comparing the concentrations obtained to the concentrations of recombinant standards run in parallel, provided in the manual's instructions.

## 5. Statistical Analysis

Results are presented as mean ± SE. Statistical significance and differences from negative and positive control values were evaluated by Students *t*-test. Statistical probability of ^*∗∗*^*P* < 0.01, ^*∗*^*P* < 0.05, and ^*∗∗∗*^*P* < 0.001 was considered to be significant.

One-way ANOVA with post hoc *t*-test was used to compare the proportion and mean values between the groups and for multiple comparisons *P* ≤ 0.05 was considered significant.

## 6. Results

### 6.1. Detection of IL-8 following the Treatment of the OMM with NE after 4- and 24-Hour Incubation

Following the treatment of the OMM with 10 nM, 50 nM, 100 nM, and 200 nM NE for 4 hours no significant results were obtained compared with the negative/positive control. However, a significant increase in the level of IL-8 was observed when comparing the OMM with 100 nM and 200 nM NE with the positive control after 24 hours (Table 1).

### 6.2. Detection of IL-1*β* following the Treatment of the OMM with NE after 4- and 24-Hour Incubation

**Figure 1:**
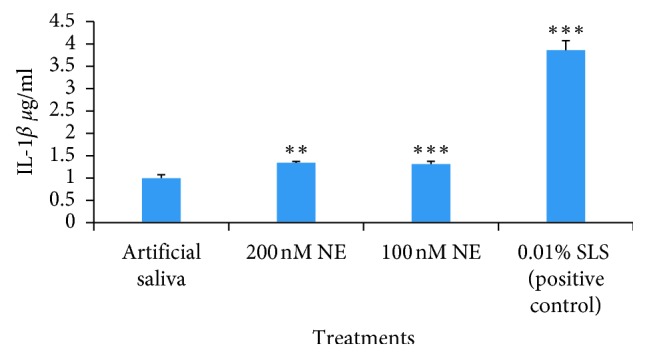
The graph depicting the concentration of IL-1*β* following the treatment of OMM samples with artificial saliva, 200 nM NE, 100 nM NE, and 0.01% SLS (*P* < 0.01^*∗∗*^, *P* < 0.001^*∗∗∗*^) for 24 hours.

**Figure 2:**
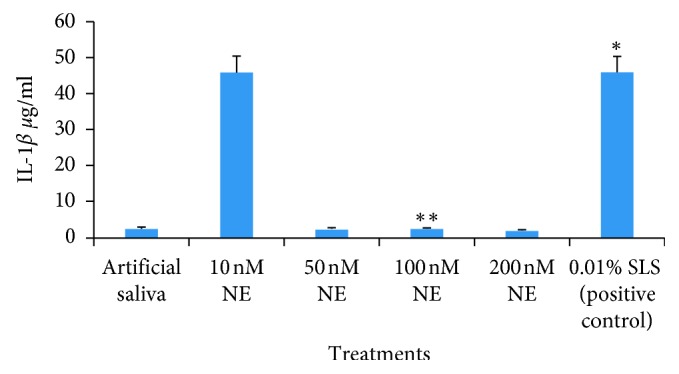
The graph depicts the concentration of IL-1*β* following the treatment of OMM samples with artificial saliva, 10 nM NE, 50 nM NE, 100 nM NE, 200 nM NE, and 0.01% SLS (*P* < 0.05^*∗*^, *P* < 0.01^*∗∗*^) after 4 hours.

A significant elevation in the level of IL-1*β* was observed following the treatment of the OMM with 100 (*P* < 0.01) and 200 nM NE after 24 hours of incubation when compared with the negative/positive control (*P* < 0.05) (Figure 1); similarly, an increased level of IL-1*β* was also observed following the treatment of OMM with 100 nM NE after 4 hours of incubation when compared with the negative/positive control (*P* < 0.05) (Figure 2) (Table 2).

### 6.3. Detection of TNF-*α* following the Treatment of the OMM with NE after 4- and 24-Hour Incubation

No significant concentration of TNF-*α* was observed following the treatment of the OMM with 10 nM, 50 nM, 100 nM, and 200 nM NE after 4- and 24-hour incubation when compared with the negative/positive control (Table 3).

## 7. Discussion

BD is a multisystem, inflammatory, and autoimmune disease [20]. The inflammatory response is orchestrated by proinflammatory cytokines such as TNF (tumor necrosis factor), interleukin-1, and IL-6 [20]. We investigated no significant increase in the level of TNF-*α* with different concentrations of neutrophil elastase after incubation for 24 hours when compared with the negative/positive control (Table 3). Similar results were observed when the OMM samples were incubated with NE of different nM concentrations for 4 h. The effect of NE on the processing of TNF-*α* is not clear, but some studies showed that NE degrades pro-TNF-*α* with loss of activity, and other investigations reveal that NE liberates biologically active TNF-*α* from its membrane-bound precursor [21]. Robache et al. investigated that protease 3 and not NE of the PNS processes TNF-*α in vitro* [22] and activates IL-8 and IL-1*β* [23].

Lorell and Eirkson have studied human neutrophil elastase for its destructive action emphysema and air flow obstruction [24]. IL-1*β* is a key mediator of inflammatory and immune responses, and monocytes are the main source of IL-1*β* [25]. We found statistically significant release of IL-1*β* when the OMM was treated with 100 and 200 nM NE and incubated for 4 h. Similarly, significant release of IL-1*β* was also observed when the OMM was treated with 100 and 200 nM NE and incubated for 24 h (*P* < 0.01) (Table 2).

Protease 3, neutrophil elastase, and cathepsin (CG) are the active neutrophil serine proteases modulating inflammatory responses by processing cytokines, growth factors, surface receptors, and signaling molecules. These NSPs not only participate in intracellular pathogenesis but also act extracellularly by degrading the protein matrix, producing chemokines and cytokines, cleaving NFƙ*β* and progranulin, and activating protease-activating receptor 2 [15].

The generalized derangement of lymphocyte and neutrophil populations was observed during the course of BD, characterized by active monocytes, increased PMN infiltration in cutaneous and ocular lesion, and increased circulating proteins, C_3–5_, IgA, hepatoglobulin, and orosomucoid. Active monocytes produce a number of proinflammatory cytokines: IL-1, IL-6, IL-8, TNF-*α*, and GM-CSF (granulocyte macrophage colony-stimulating factor). These cytokines participate in stimulating PMNL by augmented interactions with endothelial cells, causing tissue damage priming by neutrophils [18].

We examined IL-8 following the stimulation of the OMM with different concentrations of NE and found significant results (Table 1). IL-8 is a major chemokine responsible for neutrophil degranulation and neutrophil migration to inflammatory sites [25], and it is found that protease 3 [26] and not neutrophil elastase from NSPs is responsible in activating IL-8. Another study showed that NE activates the epidermal growth factor receptor [27] and induces the expression of IL-8 via toll-like receptor 4 [28] One of the studies showed that the elevated level of homocysteine in BD patients [29] is responsible for overproduction of nitric oxide from endothelial cells, induces expression of chemoattractants by oxygen free radicals, and induces IL-6, IL-8, and TNF-*α* [30, 32].

## 8. Conclusion

Saliva of patients affected with Behcet disease normally produces neutrophil elastase either with or without oral ulceration. The imbalance of neutrophil elastase causes epithelium damage and produces cytokines and chemokines, owing to prognosis of ulcer. This is the first ever study in which 3D multilayered reconstructed oral mucosal cells from oral squamous cell carcinoma were used and stimulated with different concentrations of neutrophil elastase to evaluate the releases of proinflammatory cytokines.

An elevated level of IL-1*β* was observed after 4- and 24-hour incubation with the OMM stimulated by different concentrations of NE. An increased IL-8 level was observed only after 24 hours of incubation while no TNF-*α* release was found after 4- and 24-hour incubation. So the cytotoxic effect of NE is observed in the present investigation; however, further studies are needed, and treatment of normal cell lines and patients' saliva with different concentrations of neutrophil elastase is recommended to correlate the results.

## Figures and Tables

**Table 1:** Detection of IL-8 following the treatment of the OMM with NE after 24 hours of incubation.

Parameters	NE 10 nM	NE 50 nM	NE 100 nM	NE 200 nM	Negative control (saline)	Positive control (SLS 1%)	*P* ≤ 0.05
IL-8 (pg./ml) 24 hours of incubation	—	—	2501.85^d†^ ± 610.27	2540^e†^ ± 360.27	2366.38 ± 164.08	6265.93^*∗∗∗*^ ± 356.96	ns/a†
IL-8 (pg./ml) 4 hours of incubation	202.32 ± 45.16	257.43 ± 47.33	214.34 ± 30.35	231.83 ± 57.44	189.89 ± 37.12	63.98 ± 150.50	ns/ns

^*∗*^ *P* < 0.05; ^*∗∗*^*P* < 0.01; ^*∗∗∗*^*P* < 0.001. ns = nonsignificant, a/a† = compared with the negative control/compared with the positive control, b/b† = compared the negative control with 10 nM NE/compared the positive control with 10 nM NE, c/c† = compared the negative control with 50 nM NE/compared the positive control with 50 nM NE, d/d† = compared the negative control with 100 nM NE/compared the positive control with 100 nM NE, e/e† = compared the negative control with 200 nM NE/compared the positive control with 200 nM NE, g/g† = compared 10 nM NE with 100 nM NE (negative control group)/compared 10 nM NE with 100 nM NE (positive control group), and i/i† = compared 50 nM NE with 100 nM NE (negative control group)/compared 50 nM NE with 100 nM NE (positive control group).

**Table 2:** Detection of IL-1*β* following the treatment of the OMM with NE after 24- and 4-hour incubation.

Parameters	NE 10 nM	NE 50 nM	NE 100 nM	NE 200 nM	Negative control (saline)	Positive control (SLS 1%)	*P* ≤ 0.05
IL-1*β* (pg./ml) 24 hours of incubation	—	—	1.31^d/d†^ ± 0.06	1.33^e/e†^ ± 0.02	0.99 ± 0.07	3.85^*∗∗∗*^ ± 0.21	a/a†
IL-1*β* (pg./ml) 4 hours of incubation	46.02^ns/b†^ ± 4.45	2.42^ns/c†^ ± 0.23	2.42^dgi/d†g†i†^ ± 0.21	1.92 ^e†^ ± 0.08	2.53 ± 0.25	46.05^*∗∗∗*^ ± 4.45	a/a†

^*∗*^ *P* < 0.05; ^*∗∗*^*P* < 0.01; ^*∗∗∗*^*P* < 0.001. ns = nonsignificant, a/a† = compared with the negative control/compared with the positive control, b/b† = compared the negative control with 10 nM NE/compared the positive control with 10 nM NE, c/c† = compared the negative control with 50 nM NE/compared the positive control with 50 nM NE, d/d† = compared the negative control with 100 nM NE/compared the positive control with 100 nM NE, e/e† = compared the negative control with 200 nM NE/compared the positive control with 200 nM NE, g/g† = compared 10 nM NE with 100 nM NE (negative control group)/compared 10 nM NE with 100 nM NE (positive control group), and i/i† = compared 50 nM NE with 100 nM NE (negative control group)/compared 50 nM NE with 100 nM NE (positive control group).

**Table 3:** Detection of TNF-*α* following the treatment of the OMM with NE after 24 hours of incubation.

Parameters	NE 10 nM	NE 50 nM	NE 100 nM	NE 200 nM	Negative control (saline)	Positive control (SLS 1%)	*P* ≤ 0.05
TNF-*α* (pg./ml) 24 hours of incubation	—	—	14.76 ± 1.72	23.23 ± 3.71	22.26 ± 4.09	141.84 ± 51.66	ns/ns
TNF-*α* (pg./ml) 4 hours of incubation	2.61 ± 1.43	25.00 ± 55.90	1.17 ± 0.50	4.36 ± 4.86	2.62 ± 1.64	15.29^*∗∗*^ ± 3.16	ns/ns

^*∗*^ *P* < 0.05; ^*∗∗*^*P* < 0.01; ^*∗∗∗*^*P* < 0.001. ns = nonsignificant, a/a† = compared with the negative control/compared the positive control, b/b† = compared the negative control with 10 nM NE/compared with the positive control with 10 nM NE, c/c† = compared the negative control with 50 nM NE/compared the positive control with 50 nM NE, d/d† = compared the negative control with 100 nM NE/compared the positive control with 100 nM NE, e/e† = compared the negative control with 200 nM NE/compared the positive control with 200 nM NE, g/g† = compared 10 nM NE with 100 nM NE (negative control group)/compared 10 nM NE with 100 nM NE (positive control group), and i/i† = compared 50 nM NE with 100 nM NE (negative control group)/compared 50 nM NE with 100 nM NE (positive control group).

## Data Availability

The data used to support the findings of this study are included in this article.
